# Characterizing school-linked and non-school linked SARS-CoV-2 cases in children and households: a retrospective cohort study using linked population-level administrative data from Manitoba, Canada

**DOI:** 10.23889/ijpds.v9i5.1664

**Published:** 2024-09-18

**Authors:** Jill Hnatiuk, Monica Sirski, Sharmistha Mishra, Stefan Baral, Diane Gordon-Pappas, Alan Katz

**Affiliations:** 1 Manitoba Centre for Health Policy, University of Manitoba; 2 Department of Medicine, University of Toronto; 3 Department of Epidemiology, Johns Hopkins School of Public Health

## Objective

To identify directionality of SARS-CoV-2 cases within households and schools, and characterize school, household, and neighbourhood factors associated with school and non-school linked cases.

## Objective and Approach

Using linked administrative data housed at the Manitoba Centre for Health Policy, a cohort of 449,425 individuals from 126,670 households with school-aged children enrolled in Manitoba public schools between September 1, 2020-June 30, 2021 was created. Using population-based SARS-CoV-2 laboratory test results, education enrolment and household composition data, index and transmission cases were counted and categorized at an individual and household level as school or non-school linked. Logistic regression models examined school, household and neighbourhood factors associated with school- and non-school linked households.

## Results

143280 individuals were tested for SARS-CoV-2 during the study period; 14712 individuals (10.3%) tested positive. Amongst positive cases, 1695 (11.5%) were school-linked; the remaining 13017 (88.5%) were non-school linked. 13.7% of SARS-CoV-2 positive households had ≥1 case linked to school; 86.3% did not. Preliminary results found higher incomes quintiles had lower odds of being a school-linked household, as did living outside the Winnipeg region. Having more children in a grade and more household members resulted in higher odds of being a school-linked transmission household. No associations were observed for having a child under age five in the household.

## Conclusions

Taken together, these data suggest schools were not a large source of SARS-CoV-2 cases in Manitoba and factors associated with school-linked households largely mirrored broader community transmission patterns.

## Implications

These findings may influence public health approaches to future pandemic control.

